# Dental Faculties in Medical Schools

**Published:** 1898-06

**Authors:** Richard Grady

**Affiliations:** Baltimore, Md.


### Dental Faculties in Medical Schools.

BY RICHARD GRADY, M.D., D.D.S., BALTIMORE, MD.

Presented in the Section on Stomatology, at the Forty-eighth Annual Meeting of the American Medical Association, held at Philadelphia, Pa., June 1-4, 1897.

There is a sentiment abroad that some dental schools have not the equipment of faculties and instructors that they should have. It is a prerequisite for admission to the National Association of Dental Faculties, that the applicant shall have the endorsement of the board of dental examiners of the State in which it is located; and it is the rule of the National Association of Dental Examiners that an application for recognition by that body must come from a member of the Association of Dental Faculties.

"Dental schools are not being formed now from an educational necessity. Two impulses control this matter: 1, personal ambition to have a position in and be connected with a dental school, for the prominence it is supposed to give; and 2, a purely commercial spirit on the part of medical schools. Over sixty percent of our schools are appendages to medical institutions; and nearly every dental school started in these later years has been under this outside influence."

The Committee on Colleges of the National Association of Dental Examiners, recently expressed the view that more should be required to establish the right of dental schools to recognition than the fulfillment of the rules of the Association of Dental Faculties; evidence should be furnished that the teachers are of high standing, and that they and the school have the confidence of the local members of the profession. The committee also called attention to the impropriety of schools advertising as instructors, practitioners who occasionally hold a clinic before the students, but are not a part of the staff of the institution.

While "anybody with fair mechanical ability could pull or fill a tooth, I have seen good fillings inserted in teeth by a man wholly ignorant of the science of dentistry, and we have all seen the botches which educated men have made in attempting a similar operation," yet (in the words of an editorial in the *Dental Cosmos*), "the ability to teach dentistry is an acquisition quite distinct from, though supplemental to, the ability to practice it. So important has the art of teaching become that the study and investigation of its principles now constitute a distinct branch of scientific work, viz., pedagogics. To know dentistry from the point of view of the practitioner is one thing. To know it from the teacher's standpoint implies not only all that is included in the educational equipment of the practitioner, but in addition to this the important qualification of ability to impart successfully this knowledge to others."

The plan of requirements of dental schools, hereafter making application for recognition by the National Association of Dental Examiners, provides that each dental school must have a teaching faculty of at least three professors of dental subjects, namely: for operative dentistry, for dental prothesis, and for dental pathology and therapeutics; and at least five professors for medical subjects, namely, for anatomy, physiology, chemistry, pathology and for materia medica.

Let us consider one thought in the discussion of "the equipment of faculties and instructors." In none of the dental departments of the medical schools known to us is there, in the teaching faculty, a professor for "dental pathology and therapeutics." The title used by faculties is "M.D., professor of materia medica and therapeutics," as certified to by official documents. Now, we maintain that more than a medical education (so-called) is requisite to teach dental therapeutics. The therapeutics of dentistry, unlike its anatomy, physiology and pathology, differs from that taught in the medical schools. It is medical, surgical and prosthetic. In so far as it is a direction of medical science to the prevention, modification or removal, by medicinal or hygienic remedies, of the causes and effects of disease in the dental organs, it forms part of a physician's practice, just as does the treatment of cerebral, cardiac or pulmonary disease. In so far as it is an application of surgical skill to the extraction of teeth, to the removal of tumors, to the treatment of fractures or of fissured palate, it is simply oral surgery, involving only such knowledge and skill in the use of instruments as every surgeon must possess. But dental therapeutics includes a class of operations which medical men were not taught in medical schools, and which as physicians and surgeons they do not practice. The prevailing and distinguishing feature of dental therapeutics is the art of replacement; replacement of dental structure in such manner and with such material as shall prevent further action of the destructive agencies; replacement of dental organs by substitutes which shall physiologically restore impairment of function, and esthetically restore the natural expression of the face; and this a person with a medical education alone is not qualified to teach.

